# Compassion Catalysts: Unveiling Proactive Pathways to Job Performance

**DOI:** 10.3390/bs14010057

**Published:** 2024-01-16

**Authors:** Yongjun Choi, Sung-Hoon Ko

**Affiliations:** 1 College of Business Administration, Hongik University, Seoul 04066, Republic of Korea; 2 Graduate School of Education, Kyonggi University, Suwon 16227, Republic of Korea

**Keywords:** compassion, positive emotion, job crafting, job performance, motivational approach

## Abstract

This study aims to provide a better understanding of the mechanisms underlying the relationship between the experiences of compassion and job performance. Specifically, we test if positive emotion and job crafting could help explain the experience of the compassion–job performance link. Using a sample of 312 employees in large-sized domestic companies located in South Korea, we found that the experience of compassion was positively related to job performance. In addition, we adopted the motivational approach to demonstrate that the positive relationship between the experience of compassion and job performance was sequentially mediated by positive emotion and job crafting. Our study advances the literature on workplace compassion by introducing job crafting as a novel driver in explaining the positive effects of compassion and contributes novel insights into the mechanisms underlying the relationship between compassion and job performance. Our findings also suggest that to enhance employees’ job performance and facilitate employees’ proactive behaviors (i.e., job crafting), organizations must foster a compassionate work environment by placing high importance on compassion.

## 1. Introduction

Experiences of suffering, i.e., “distressing felt experience that affects one’s fundamental intactness as a person” [1] (p. 85), at work is a significant and inevitable aspect of organizational lives [2,3,4,5]. In this vein, similar to suffering at work, Kanov [1] argues that compassion should also be viewed as a building block of organizations that is relevant in all organizations, because receiving compassion from others (i.e., experiencing others’ expression of concerns and caring toward one’s suffering) is integral to alleviating one’s experiences of suffering [2,5]. Because the experiences of suffering could drain one’s personal resources [6], which is detrimental for work motivation [7,8], it is rather likely that receiving compassion from others at work could not only alleviate one’s suffering but also stimulate one’s work motivation which, in turn, leads to higher levels of job performance. Specifically, given that receiving genuine care from organizations, such as compassion and helping behaviors from coworkers, increases intrinsic motivation for proactive behaviors, such as creativity [9,10,11], it is more likely that an individual’s proactivity could explain the link between compassion and job performance.

A growing body of research has explored the roles of compassion in organizations and showed its positive effects on work outcomes [2,12]. For instance, previous studies have shown that employees who receive compassion from their coworkers are likely to experience more positive emotion [13,14,15,16,17], be more emotionally committed to their organizations [5], and even display higher levels of job performance [18,19,20,21]. More importantly, previous research has consistently revealed a positive relationship between compassion and job performance. Specifically, regarding the compassion–job performance link, past studies have attempted to unbox the mechanisms and demonstrate the mediating roles of positive emotion [22], positive work-related identity [20,23], and collective self-esteem [23]. In other words, they have elucidated that as a result of receiving compassion from others, employees experience positive emotion and shape positive attitudes which, in turn, leads to high job performance.

In spite of the progress made in the literature, our knowledge of why and through what mechanisms compassion leads to high job performance is still in a nascent state [1,24]. One of the important antecedents for job performance is proactivity, because proactive individuals create and select situations, such as creating work environments for accentuating their strengths [25], that are most likely to deliver high levels of performance [26]. Past studies on organizational socialization have also evinced that proactive employees engage in a wide range of instrumental behaviors, such as information and feedback-seeking, sensemaking, and role restructuring, which are critical for job performance [27,28,29,30,31]. Thus, given that the experiences of compassion could help replenish one’s interpersonal sources [32], which is vital for job performance [33,34], it is worth exploring the roles of proactivity in the compassion–job performance link. Specifically, we focus on the role of job crafting, because it captures employees’ proactive behaviors of shaping the boundaries of their work and creating work environments that best suit their competencies [35], which, in turn, leads to higher job performance [36].

In this study, we seek to elucidate the mechanisms through which the experiences of compassion at work increase one’s job performance. Specifically, our study aims to advance existing knowledge by exploring the interplay between compassion and job performance, specifically through the lens of job crafting. The JD-R model [37,38,39] serves as the overarching framework for our research on compassion and job performance. Compassion is viewed as a job resource aiding employees in managing emotional work demands. Positive emotion and job crafting are mechanisms that mediate the relationship between compassion and job performance. Positive emotion can be considered a personal resource, while job crafting is a proactive behavior that involves employees shaping and redesigning their own jobs to make them more meaningful and engaging. This study proposes a sequential mediation model, indicating that the positive relationship between compassion and job performance is sequentially mediated by positive emotion and job crafting, aligning with the JD-R theory’s idea that job resources can lead to positive outcomes sequentially.

Our study makes several contributions to the literature on workplace compassion. First, through our focus on the serial mediation effects of positive emotion and job crafting on the relationship between compassion and job performance, we extend the findings of Chu [22], who demonstrated the mediating roles of positive emotion in the compassion–job performance link. Given that we currently have somewhat limited knowledge about the mechanisms underlying compassion and job performance [1,24], our findings provide a deeper understanding of how the experiences of compassion at work translate into job performance. Second, we incorporate the motivational approach in examining the relationship between compassion and job performance by introducing and testing the roles of job crafting. Although prior research on workplace compassion has primarily focused on its effects on one’s emotions [5,13,17] or cognitive attitudes [20,23], our findings provide new insights into the literature on compassion by highlighting the motivational processes that are triggered by the experiences of compassion at work.

## 2. Hypotheses Development

Compassion is a multidimensional process encompassing noticing others’ suffering, feeling empathy toward them, and acting to alleviate their suffering [3,5,14,40,41]. Employees who experience compassion at work are likely to perceive that they are being genuinely cared for by others, helping them reinforce the connections between employees. Thus, based on the social exchange theory [42], compassion at work may serve to maintain positive exchange relationships with others, increasing the positive exchanges with others [5] and allowing them to have more opportunities for learning [43], which, in turn, increases job performance. Likewise, according to the job demands–resources (JD-R) model [37,38,39], experiencing compassion is likely to serve as an interpersonal resource [32], helping employees better fulfill their job requirements, and subsequently leading to high job performance. Previous research has also supported the idea that experiencing compassion at work increases one’s job performance [44] via, for example, increasing one’s positive moods [22], a positive work-related identity [20], and thriving at work [19]. Therefore, building upon the past findings, we hypothesize that experiencing compassion at work will increase an employee’s job performance.

**Hypothesis 1.**  *The compassion experienced by employees is positively related to job performance.*

The affective events theory [45] (Weiss and Cropanzano 1996) suggests that work events trigger employees’ emotional reactions which, in turn, affects their job satisfaction and job performance. Compassion at work can serve as important events generating employees’ positive emotions, because it entails an act of mental and physical care for those who experience suffering at work [22]. Furthermore, receiving compassion at work could strengthen the emotional relationship quality among employees, helping induce positive emotions [40]. The JD-R model [37,38,39] also supports the link between compassion and positive emotion, because compassion coming from supervisors and colleagues can help employees better manage their work stresses and job demands by replenishing their emotional resources [15,32], which are closely related to positive emotions. Previous research on compassion has provided support for the positive link between compassion and positive emotions [13,14,15,16,17]. For example, the seminal work by Lilius et al. [5] demonstrates that employees receiving compassion from others experience positive emotions, such as joy, inspiration/encouragement, comfort, and pride, as a result of feeling connectedness and belonging within organizations. Thus, we hypothesize that experiencing compassion at work will increase an employee’s positive emotions.

**Hypothesis 2.**  *The compassion experienced by employees is positively related to positive emotions.*

The broaden–build theory suggests that positive emotions serve a unique function in helping individuals recover from challenging situations via the generation of emotional resources [46], as well as by expanding one’s cognition and thought repertoire [7,46]. In support of Fredrickson [7,46], Avey et al. [47] argue that positive emotions expand the path toward goals and help individuals regard the challenges arising from this goal-oriented process as an extrinsic, insignificant, and temporary phenomenon. Thus, we can expect that employees who experience positive emotions are more likely to engage in proactive behaviors at work, which is “taking initiative in improving current circumstances or creating new ones” [25] (p. 436). This is also congruent with previous studies suggesting that one’s motivation to engage in proactive behaviors at work is, in part, influenced by one’s emotional states at work [45,48,49].

Among several proactive behaviors at work, we expect that positive emotions could facilitate job crafting. Job crafting, which refers to “the physical and cognitive changes individuals make in the task or relational boundaries of their work” [35] (p. 179), is an important self-oriented proactive behavior at work. Positive emotions can help employees identify their strengths and increase their autonomy in decision making [50], thereby facilitating their generation of creative ideas and actions to better perform at their jobs [51]. In addition, as the broaden-and-build theory suggests [7,46], employees’ attention and thinking are broadened by positive emotions, facilitating greater connections and wider-range ideas [52,53] and enabling them to be more open to new experiences [52,54]. That is, because positive emotions help the construction of personal resources [55], which are essential for self-directed work design (i.e., job crafting), it is more likely that positive emotions energize one’s work-related creativity which, in turn, allows one to transform the status quo into more favorable work conditions. As such, we hypothesize that employees who experience positive emotions are more likely to engage in job crafting.

**Hypothesis 3.**  *Positive emotion is positively related to job crafting.*

The meta-synthesis by Lazazzara et al. [56] identified that improving job performance is one of the major motivations for job crafting. That is, employees who engage in job crafting proactively redesign their own jobs, because they believe that those behaviors could help improve their job performance via enhancing their self-image and sense of control [57]. Applying the JD-R model [37,38,39] to job crafting [35], Tims et al. [58] suggest that job crafting increases job resources and challenging job demands while decreasing hindering job demands. That is, by crafting their job characteristics, employees can create more resourceful working environments which, in turn, increase their job performance. Ample evidence also provides support for the positive link between job crafting and job performance [36,58,59,60]. For example, Tims et al. [58] find that the more employees who engaged in job crafting, the more favorably their supervisors rated their performance. In addition, Tims et al. [58] and Shin et al. [60] have reported that job crafting leads to high levels of job performance via the enhancement of work engagement. In a similar vein, a recent meta-analysis by Rudolph et al. [61] also demonstrates that overall job crafting is positively related to both self-rated and other-rated work performance. Therefore, we expect the following:

**Hypothesis 4.**  *Job crafting is positively related to job performance.*

A few studies have explored the mechanisms through which the experiences of compassion at work could increase one’s job performance [19,20,22,23]. One stream of the previous studies adopted a cognitive approach to unbox the relationship between compassion and job performance. For instance, Hur et al. [20] show that a positive work-related identity plays a key role in the link between compassion and job performance. That is, based on the social identity theory [62,63], they demonstrate that service employees who experience compassion at work perceive that they are treated genuinely and valued favorably by others, leading to the development of a positive work-related identity that allows them to achieve high levels of performance. Another stream of previous research adopted an affective approach to explore the paths through which compassion leads to an increase in job performance. For instance, building upon Lilius et al. [5] and previous research on emotions, Chu [22] shows that registered nurses who experience positive emotions as a result of experiencing compassion at work increase their work motivation, leading to high levels of job performance. However, our knowledge of the underlying mechanisms about the link between compassion and job performance is still nascent. Therefore, to elucidate the underlying paths for the positive relationship between compassion and job performance, we propose positive emotion and job crafting as possible double-mediating variables.

Drawing mainly upon the affective events theory [45] and the JD-R model [37,38,39], this study suggests that experiencing compassion at work not only directly but also indirectly influences job performance via the increases in positive emotion and job crafting. Experiencing suffering at work, arising from many different sources, such as toxic interactions with organizational insiders and customers, is an inevitable part of organizational lives [5]. Thus, receiving compassion from others at work is interpreted as critical, as are positive work events evoking positive emotions [22], because although suffering could drain personal resources, the experience of compassion could replenish one’s personal resources, which are vital for facilitating healthy behaviors [6]. Specifically, in the context of this present study, thanks to the positive emotions, employees with personal resources are very likely to take initiatives to change the boundaries of their jobs, such as seeking better and creative ideas for increasing their performance [51], which, in turn, increases one’s job performance. It is also congruent with Bono and Ilies [64], who contend that experiencing positive emotions is one of the fundamental psychological processes for creating productive performance. Therefore, we hypothesize as follows:

**Hypothesis 5.**  *The positive relationship between compassion and job performance is serially mediated by positive emotion and job crafting.*

**Figure 1:**
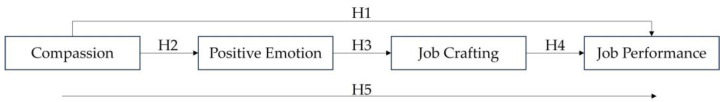
Research model.

Our research model is presented in Figure 1.

## 3. Methods

### 3.1. Participants and Procedure

Participants in this study were full-time employees at large-sized domestic companies located in South Korea, with workforce sizes ranging between approximately 7000 and 120,000 employees. Specifically, participants were those who work in teams that require self-directed and/or creative tasks. The number of participants from each company ranged from 22 to 39. We used the survey method to collect data from study participants. A member of the research team visited ten large-sized domestic companies and gave presentations to the potential participants to increase the participation rate. A total of 350 employees expressed that they were willing to voluntarily participate in the survey. Thus, 350 surveys were distributed; the potential participants were also given a link to an online version of the survey so that they could participate in the survey either offline or online. Among those 350 potential participants, a total of 330 participated in the survey, resulting in a response rate of 97.1%. Excluding 18 surveys with insincere responses (e.g., responses with a central tendency bias), the study finally entered 312 surveys into data analysis. In the context of this study, insincere responses denote instances where respondents disengage with survey items. This disengagement is characterized by a consistent selection of identical numerical values across all items, indicating a centralization tendency. Additionally, insincere responses include situations where respondents omit answers to multiple questions within the survey. Of the 312 participants, 182 (58.3%) were female. The majority of participants were in their 30 s (41.7%) and 40 s (29.2%) and had tenured for 1–4 years (43.6%) or 5–9 years (26.6%).

### 3.2. Measurement

The Cronbach’s α values reported below are derived from our sample of 312 participants.

Compassion. Three items developed by Lilius et al. [5] were used to measure the experience of compassion at work: “I often experience compassion at my workplace”, “I often experience compassion from my supervisor”, and “I often experience compassion from my colleagues”. Responses for the items ranged from 1 (strongly disagree) to 5 (strongly agree), and Cronbach’s α was 0.894.

Positive Emotion. Positive emotion was defined as one’s positive emotional state resulting from one’s experiences within an organization. Four items from Lilius et al. [5] were used. Sample items include “I am proud of all my work” and “I am happy with my work”. The items were rated on a 5-point Likert scale (1 = strongly disagree to 5 = strongly agree), and Cronbach’s α was 0.916.

Job Crafting. Job crafting was defined as employees’ self-directed handling and redesigning of their jobs in their own ways. Four items from Wrzesniewski and Dutton [35] and Morrison and Phelps [65] were used. Sample items are “I find a new method of my own to improve the work in the organization” and “I change the way I work myself to make the work easier”. Responses for the items ranged from 1 = strongly disagree to 5 = strongly agree, and Cronbach’s α was 0.808.

Job Performance. Job performance was defined as the degree of responsibly completing one’s assigned work. Five items from Williams and Anderson [66] were used. Sample items include “I adequately complete assigned duties” and “I fulfill responsibilities that are expected of me”. Responses ranged from 1 = strongly disagree to 5 = strongly agree, and Cronbach’s α was 0.900.

Control Variables. We also measured basic demographic information about the participants including gender, age, position, and tenure. Among the four demographic variables, only age was statistically significantly related to our study variables. For example, age was positively and significantly related to job crafting (r = 0.138, *p* < 0.05). This is consistent with previous studies showing that younger and older people have different motivations and preferences for job crafting interventions [67,68]. Thus, following the recommendations for the use of control variables [69,70,71], among the demographic variables, we retained only the age, in an attempt to control their possible influence on compassion, positive emotion, job crafting, and job performance.

For the full list of items used in this study, please refer to Appendix A. In addition, the result of composite reliability is presented in Table 1.

### 3.3. Testing of Common Method Bias

This study collected data from the same respondents at a single point in time, raising an issue about a potential common method bias. Hence, we conducted a Harman’s one-factor test as an ex-post remedy to see if the measurement involves a common method bias issue [72]. If the total variance extracted by a single factor surpasses 50%, it indicates the presence of common method bias. Four factors with an eigenvalue greater than one were found, and the covariance explained by one factor (before being rotated) was 18%. Thus, we concluded that the common method bias was not a serious concern in this study.

We further conducted the latent method factor control approach incorporating the significance of *χ*^2^ changes in relation to the changes in degrees of freedom, model fit, and estimation, along with the statistical significance of the path coefficient [72,73]. In essence, a significant change in *χ*^2^ relative to changes in degrees of freedom, when comparing pre- and post-control for potential method factors, indicates the existence of common method bias.

The results are presented in Table 2. The model fit before control was *χ*^2^ = 141.270 (df = 95, *p* = 0.000), CFI = 0.985, TLI = 0.981, IFI = 0.985, RMSEA = 0.040, and RMR = 0.022. After controlling for latent variables, the model fit was *χ*^2^ = 112.664 (df = 84, *p* = 0.000), CFI = 0.989, TLI = 0.984, IFI = 0.989, RMSEA = 0.037, and RMR = 0.015. A comparative analysis of the two models indicated a non-significant difference in Δ value (28.606, *p* > 0.05), which is attributable to the difference in degrees of freedom (df = 11). This observation suggests that common method bias does not significantly affect our model. Additionally, as depicted in Table 3, the absolute values of the λ-CMV values—indicating the difference between the λ value before and after control—do not surpass 0.2. Following the criteria proposed by Williams et al. [73] and Podsakoff et al. [72], this suggests a low likelihood of common method bias between the latent variables used in our study model.

## 4. Results

The descriptive statistics and bivariate correlations for our study variables are presented in Table 4. Compassion was positively and significantly related to positive emotion (r = 0.356, *p* < 0.01) and job performance (r = 0.361, *p* < 0.01). Positive emotion (r = 0.289, *p* < 0.1) and job crafting (r = 0.658, *p* < 0.01) were positively and significantly related to job performance. The study also ran a regression analysis to examine multicollinearity, and the results showed VIFs ranging from 1.112 to 1.268, demonstrating no serious multicollinearity issue.

We conducted a confirmatory factor analysis (CFA) to establish the discriminant validity and test the goodness-of-fit of our research model before testing our study hypotheses. The CFA results indicated an acceptable fit (CFI = 0.985; TLI = 0.981; GFI = 0.947; IFI = 0.985; RMSEA = 0.040; RMR = 0.022). Furthermore, the average variance extracted (AVE) for each of the four study constructs was greater than 0.6, confirming the discriminant validity, while Cronbach’s alpha for each construct was greater than 0.8.

We tested our study hypotheses from 1 to 4 by conducting structural equation modeling using AMOS 24.0. Specifically, we examined the path coefficients from the structural model. In support of Hypothesis 1, the path from compassion to job performance was positive and statistically significant (β = 0.121, SE = 0.041, CR = 2.921, *p* < 0.01). In support of Hypothesis 2, the path from compassion to positive emotion was also positive and statistically significant (β = 0.337, SE = 0.053, CR = 6.329, *p* < 0.001). In addition, the path from positive emotion to job crafting was positive and statistically significant (β = 0.262, SE = 0.052, CR = 5.085, *p* < 0.001), which provides support for Hypothesis 3. Hypothesis 4, which is about the positive relationship between job crafting and job performance, was also supported, because the path coefficient was positive and statistically significant (β = 0.581, SE = 0.043, CR = 13.511, *p* < 0.001). The path coefficients for the study hypotheses are presented in Table 5.

Hypothesis 5 suggests the serial mediation effects of positive emotion and job crafting in the relationship between compassion and job performance. For testing the indirect effect, we implemented the bootstrapping method with 5000 iterations [74,75]. Specifically, in addition to calculating the estimates, we obtained a 95% bias-corrected confidence interval (CI) to determine if the mediating variables can help explain the relationship between an independent and dependent variable in our study. The bootstrapped results are presented in Table 6. Compassion was positively and statistically significantly related to job performance via positive emotion (indirect effect = 0.020, CI = [0.014, 0.056]), as the 95% CI does not include zero. In addition, the indirect effect of compassion on job performance was also positive and statistically significant (indirect effect = 0.148, CI = [0.075, 0.221]), as the 95% CI does not include zero. Finally, the bootstrapping results show that positive emotion and job crafting serially mediate the relationship between compassion and job performance (indirect effect = 0.034, CI = [0.008, 0.068]), as the 95% CI does not include zero. Hence, Hypothesis 5 was supported.

## 5. Discussion

### 5.1. General Discussion

Although the roles of compassion at work in facilitating employees’ positive work attitudes and behaviors have received increasing attention from researchers [2,12,15], there is less clarity regarding the mechanisms of experiences of compassion that lead to high job performance [1,24]. Thus, the major goal of this present study was to unbox the mechanisms underlying the relationship between compassion and job performance. Therefore, we investigated employees’ positive emotions and job crafting as critical mediators that provide explanations of why this effect exists. As predicted, we observed positive relationships, indicating that when employees experience compassion at work, there is a simultaneous increase in positive emotions which, in turn, appears to be linked to a tendency to engage in job crafting behaviors, which contributes to enhanced job performance. By introducing and investigating the mediating role of job crafting in the compassion–job performance relationship, our findings extend previous knowledge about the mediating role of positive emotion in the compassion–job performance link [22]. Additionally, this study offers new insights into the motivational processes that elucidate the roles of compassion in the workplace.

### 5.2. Theoretical Implications

The findings from this current study advance current theories in the following ways: First, building on the findings of Chu [22], who showed that positive emotions mediate the relationship between compassion and job performance, we demonstrated that job crafting behaviors could even help elucidate the underlying mechanism. Besides Chu [22], prior studies have primarily focused on an individual’s cognitive responses as a result of their experiences of compassion at work. For instance, grounded in the social identity theory [62,63], Hur et al. [20] and Ko and Choi [23] have shown that employees who experience compassion at work are likely to construct their identities congruently with their organizations, increasing their intrinsic motivation to accomplish higher job performance. Hence, our approach offers a much deeper understanding of the affective aspect of how experiencing compassion could increase one’s job performance.

Second, our findings contribute to the current literature on workplace compassion and job crafting by integrating the motivational approach. Central to our theory is the mediating role of job crafting, as one form of proactive behaviors at work, in the compassion–job performance link. Although Hur et al. [20] suggest that the experiences of compassion could increase one’s intrinsic motivation, unfortunately, they have not explicitly tested if receiving compassion from others actually triggers one’s motivational work processes. In this study, our findings indicate an association wherein employees’ positive emotions, arising from the experience of compassion, are linked to the facilitation of their proactive behaviors (i.e., job crafting), because positive emotions help the construction of and bring more personal resources [76] that are vital for taking proactive initiatives at work. Likewise, it is congruent with the past findings that employees who experience positive emotions are likely to experience higher levels of resilience [77]. That is, because employees are highly likely to experience suffering at work, experiencing compassion could be associated with a potential for successful recovery from work-related sufferings. This association, in turn, is linked to the experience of positive emotions, which appears to be associated with heightened work motivation (i.e., job crafting) and, consequently, high levels of job performance. In addition, our study introduces a new outcome variable (i.e., job crafting) of workplace compassion. Although Chu [22] demonstrates that experiencing compassion could increase organizational citizenship behavior, as one forms proactive behaviors, to the best of our knowledge, the effects of compassion on job crafting have not been explored so far. In a similar vein, the roles of compassion have not been tested in the past literature on job crafting. Therefore, our motivational approach toward workplace compassion, with an emphasis on job crafting, advances the current literature on workplace compassion and job crafting.

### 5.3. Practical Implications

Our study provides practical implications for organizations. Our findings indicate that organizations can foster employees’ job crafting by providing them with the experiences of compassion at work. In general, our findings also imply that compassionate experiences at work could energize employees’ proactive behaviors. Given that employees’ proactive behaviors, such as job crafting, are critical for job performance [36,56,58,59,60], which is also consistent with our findings, creating a compassionate environment at work could help the organizations better develop their human resources [78,79]. For this, organizations could provide the employees with opportunities or formal programs to identify and alleviate others’ suffering in the workplace. For instance, organizations could train their employees in how to communicate with others in a compassionate manner [80] or help them develop and enact compassionate leadership [81]. Organizations may also integrate compassion as a core value within their mission and vision statements. Moreover, leaders are instrumental in shaping a culture that prioritizes empathy and understanding by actively endorsing and exemplifying compassionate behaviors. To reinforce this commitment, organizations can also establish policies and practices that prioritize employee well-being. For instance, the establishment of Employee Assistance Programs (EAPs) offers support to individuals navigating personal or professional challenges, signaling an organization’s dedicated commitment to employee well-being. Additionally, organizations can foster mentorship programs and promote peer support initiatives, creating a network where employees feel at ease seeking guidance and assistance from their colleagues. This strategic emphasis on compassion could contribute to the development of a workplace environment that values compassion.

### 5.4. Limitations and Future Research Directions

Despite the theoretical and practical implications, this study has the following limitations that warrant future research: First, our cross-sectional research design using single-source data raises potential common method bias. Specifically, although we hypothesized and tested the causal link between positive emotion and job crafting, as Slemp et al. [82] evince, it is also feasible that job crafting induces employees’ positive emotions. In addition, even though we concluded that a common method variance is not a serious concern by conducting Harman’s one-factor test, we acknowledge that we cannot completely rule out of the possibility of common method variance in our model. Especially, the use of a self-reported job performance measure could potentially inflate our findings. Thus, future research is encouraged to further validate our findings by using a longitudinal research design as well as multisource data. Specifically, future research would benefit from obtaining objective performance measures from sources such as employees’ annual performance reviews.

Second, our study focused on the effect of receiving compassion in the workplace. Although most studies on workplace compassion have shown the positive effects of receiving compassion, previous studies, especially focusing on nurses, health care employees, or social workers, highlighted the negative effects of providing compassion, such as compassion distress or compassion fatigue [83,84,85,86]. However, a recent study by Chu [18] presents conflicting results; using a sample of 235 nurses in Taiwan, Chu [18] demonstrates that similar to those who receive compassion at work, compassion givers can also experience increases in job performance and mental health as a result of fulfilling their basic human needs such as social relatedness [87]. Thus, future research could benefit by replicating our theoretical model from a compassion giver’s perspective and see if the more an individual provides compassion to others, the more employees engage in proactive behaviors, such as job crafting.

Third, although we have examined the roles of experiencing compassion at work, we did not explicitly explore the factors that could facilitate employees’ experiences of compassion. However, the practical implications drawn from our findings suggest a potentially close interconnection among organizations, leaders, and employees, indicating the likelihood of trickle-down effects wherein organizations influence leaders, leaders influence employees, and organizations may even have a direct impact on employees. In essence, considering the significant effects of organizations and leadership on compassion, positive emotion, and job crafting, future research may benefit from adopting a multilevel analysis approach [88]. Investigating how these constructs operate at both the individual and organizational levels can provide a more comprehensive understanding of the complex interplay between compassion, job crafting, and job performance.

Fourth, this present study focused on job crafting as one form of proactive behavior in the workplace. However, because proactive behaviors in general also require one’s use of personal resources [55,76], beyond job crafting, it is feasible that employees who experience compassion are likely to engage in other forms of proactive behaviors at work. Thus, extending our findings, future research could explore if the experiences of compassion could enhance other forms of proactive behaviors, such as proactive strategic behaviors and proactive person–environment fit behaviors [89,90].

Fifth, while it is common to measure job crafting through surveys, we recognize the limitations associated with relying solely on survey methods and statistical analysis in our study, as highlighted by Wrzesniewski and Dutton [35]. Scholars have conducted qualitative studies to better understand job crafting dynamics [91,92], and some have adopted mixed methods to examine these phenomena [93]. Similarly, qualitative studies have explored the phenomenon of compassion at work [94,95]. Recognizing the benefits of a mixed-methods design for a more comprehensive exploration of the complexities surrounding compassion, job crafting, and their interplay, we acknowledge that such approaches would enable a more thorough understanding of the phenomena. Hence, future research would benefit from expanding the research design and methodology to incorporate a mixed-methods study.

## 6. Conclusions

Our study has unveiled the relationship between workplace compassion and job performance, emphasizing the pivotal roles of positive emotions and job crafting as mediating factors. Compassion, by triggering positive emotions, stimulates job crafting behaviors, ultimately leading to improved job performance. This underscores the significance of nurturing a compassionate workplace atmosphere to fuel employees’ proactive behaviors, particularly through job crafting, with the overarching goal of enhancing organizational performance. Our research provides valuable insights into the concrete advantages of compassion in the workplace, urging organizations to adopt compassionate practices to cultivate a more productive and harmonious work environment.

## Figures and Tables

**Table 1:** Result of composite reliability.

Construct	Items	AVE	α	C.R
Compassion	Com1	0.810	0.894	0.940
	Com2			
	Com3			
Positive Emotion	PE1	0.813	0.916	0.947
	PE2			
	PE3			
	PE4			
Job Crafting	JC1	0.786	0.808	0.864
	JC2			
	JC3			
	JC4			
Job Performance	JP1	0.843	0.900	0.939
	JP2			
	JP3			
	JP4			
	JP5			

Note: Com = compassion; PE = positive emotion; JC = job crafting; JP = job performance.

**Table 2:** Analysis of common method bias.

	*χ* ^2^	df	*p*	*χ*^2^/df	RMSEA	CFI	NFI	IFI	TLI
Measurement Model (M.M)	141.270	95	<0.001	1.487	0.040	0.985	0.957	0.985	0.981
Controlled Model (C.M.)	112.664	84	<0.001	1.341	0.037	0.989	0.965	0.989	0.984
Stepwise Analysis	Δχ2	Δdf		Accepted Model					
M.M.-C.M.	28.606	11	>0.05	Measurement Model					

**Table 3:** Analysis of common method bias: absolute λ-CMV differences.

Construct	Items	λ	λ (CMV)	λ-λ (CMV)	SE	CR	AVE	α	C.R
Compassion	Com1	0.853	0.725	0.128	-	-	0.810	0.894	0.940
	Com2	0.882	0.709	0.173	0.057	18.599			
	Com3	0.843	0.743	0.100	0.057	17.750			
Positive Emotion	PE1	0.831	0.800	0.031	-	-	0.813	0.916	0.947
	PE2	0.788	0.747	0.041	0.046	20.412			
	PE3	0.871	0.814	0.057	0.058	17.997			
	PE4	0.881	0.792	0.089	0.062	18.182			
Job Crafting	JC1	0.794	0.711	0.083	-	-	0.786	0.808	0.864
	JC2	0.685	0.661	0.024	0.081	11.291			
	JC3	0.741	0.679	0.062	0.076	12.326			
	JC4	0.564	0.443	0.121	0.083	9.429			
Job Performance	JP1	0.830	0.739	0.091	-	-	0.843	0.900	0.939
	JP2	0.805	0.719	0.086	0.051	19.712			
	JP3	0.803	0.697	0.106	0.060	15.920			
	JP4	0.813	0.756	0.057	0.060	16.203			
	JP5	0.726	0.627	0.099	0.065	13.960			
*χ*^2^ = 112.664 (df = 79, *p* = 0.000), CFI = 0.989, TLI = 0.984, IFI = 0.989, NFI = 0.965, RMSEA = 0.037, RMR = 0.015									

Note: Com = compassion; PE = positive emotion; JC = job crafting; JP = job performance.

**Table 4:** Means, standard deviations, and correlations.

	Mean	SD	1	2	3	4
1. Compassion	3.433	0.706	0.810			
2. Positive Emotion	3.229	0.707	0.356 **	0.813		
3. Job Crafting	3.356	0.667	0.350 **	0.301 **	0.786	
4. Job Performance	3.682	0.654	0.361 **	0.289 **	0.658 **	0.843

Note: *N* = 312; ** *p* < 0.01; the numbers along the diagonal are the square root of the averaged variance extracted (AVE).

**Table 5:** Path coefficients for hypotheses.

Hypotheses	Path	β	SE	CR	*p*
Hypothesis 1	Compassion ⟶ Job Performance	0.121	0.041	2.921	<0.01
Hypothesis 2	Compassion ⟶ Positive Emotion	0.337	0.053	6.329	<0.001
Hypothesis 3	Positive Emotion ⟶ Job Crafting	0.262	0.052	5.085	<0.001
Hypothesis 4	Job Crafting ⟶ Job Performance	0.581	0.043	13.511	<0.001

**Table 6:** Indirect effect of the double-mediation effects (positive emotion and job crafting).

From ⟶ To	Indirect Effect		
	Estimate	CI_low_	CI_high_
Total indirect effect	0.202	0.117	0.286
Comp ⟶ PE ⟶ JP	0.020	0.014	0.056
Comp ⟶ JC ⟶ JP	0.148	0.075	0.221
Comp ⟶ PE ⟶ JC ⟶ JP	0.034	0.008	0.068

Note: *N* = 312; bootstrap confidence intervals (CIs) were obtained based on 5000 resamples; Comp = compassion; PE = positive emotion; JC = job crafting; JP = job performance.

## Data Availability

The data presented in this study are available on request from the corresponding author.

## Appendix A. Survey Instrument and Measurement Scales

Compassion

1. I frequently experience compassion on the job.
2. I frequently experience compassion from my supervisor.
3. I frequently experience compassion my co-workers.

Positive Emotion

1. I often feel proud within the organization.
2. I often feel grateful within the organization.
3. I often feel inspired within the organization.
4. I often feel at ease within the organization.

Job Crafting

1. I often make constructive suggestions for improving how things operate within the organization.
2. I often try to implement solutions pressing organizational problems.
3. I often try to introduce new structures, technologies, or approaches to improve efficiency.
4. I often try to institute new work methods that are more effective for the company.

Job Performance

1. I adequately complete assigned duties.
2. I fulfill responsibilities specified in job description.
3. I meet formal performance requirements of the job.
4. I engage in activities that will directly affect his/her performance evaluation.
5. I perform task that are expected of him/her.

## References

[1] Kanov J. (2021). Why suffering matters!. J. Manag. Inq..

[2] Dutton J.E., Workman K.M., Hardin A.E. (2014). Compassion at work. Annu. Rev. Organ. Psychol. Organ. Behav..

[3] Dutton J.E., Worline M.C., Frost P.J., Lilius J.M. (2006). Explaining compassion organizing. Adm. Sci. Q..

[4] Frost P.J. (1999). Why compassion counts!. J. Manag. Inq..

[5] Lilius J.M., Worline M.C., Maitlis S., Kanov J., Dutton J.E., Frost P. (2008). The contours and consequences of compassion at work. J. Organ. Behav..

[6] Fredrickson B.L. (2000). Cultivating positive emotions to optimize health and well-being. Prev. Treat..

[7] Fredrickson B.L. (1998). What good are positive emotions?. Rev. Gen. Psychol..

[8] Salanova M., Bakker A.B., Llorens S. (2006). Flow at work: Evidence for an upward spiral of personal and organizational resources. J. Happiness Stud..

[9] Amabile T.M., Staw B.M., Cummings L.L. (1988). A model of creativity and innovation in organizations. Research in Organizational Behavior.

[10] Gagné M., Deci E.L. (2005). Self-determination theory and work motivation. J. Organ. Behav..

[11] Hur W.-M., Moon T.-W., Ko S.-H. (2018). How employees’ perceptions of CSR increase employee creativity: Mediating mechanisms of compassion at work and intrinsic motivation. J. Bus. Ethics.

[12] Worline M.C., Dutton J.E. (2017). Awakening Compassion at Work: The Quiet Power That Elevates People and Organizations.

[13] Dutton J., Frost P., Worline M., Lilius J., Kanov J. (2002). Leading in times of trauma. Harv. Bus. Rev..

[14] Dutton J.E. (2003). Energize Your Workplace: How to Create and Sustain High-Quality Connections at Work.

[15] Frost P. (2003). Toxic Emotions at Work: How Compassionate Managers Handle Pain and Conflict.

[16] Kahn W.A. (1993). Caring for the caregivers: Patterns of organizational caregiving. Adm. Sci. Q..

[17] Ko S.-H., Kim J., Choi Y. (2021). Compassion and workplace incivility: Implications for open innovation. J. Open Innov. Technol. Mark. Complex..

[18] Chu L.C. (2017). Impact of providing compassion on job performance and mental health: The moderating effect of interpersonal relationship quality. J. Nurs. Scholarsh..

[19] Elahi N.S., Abid G., Arya B., Farooqi S. (2020). Workplace behavioral antecedents of job performance: Mediating role of thriving. Serv. Ind. J..

[20] Hur W.-M., Moon T., Rhee S.-Y. (2016). Exploring the relationships between compassion at work, the evaluative perspective of positive work-related identity, service employee creativity, and job performance. J. Serv. Mark..

[21] Moon T.-W., Hur W.-M., Ko S.-H., Kim J.-W., Yoo D.-K. (2016). Positive work-related identity as a mediator of the relationship between compassion at work and employee outcomes. Hum. Factors Ergon. Manuf. Serv. Ind..

[22] Chu L.C. (2016). Mediating positive moods: The impact of experiencing compassion at work. J. Nurs. Manag..

[23] Ko S.-H., Choi Y. (2019). Compassion and job performance: Dual-paths through positive work-related identity, collective self esteem, and positive psychological capital. Sustainability.

[24] Frost P.J., Dutton J.E., Maitlis S., Lilius J.M., Kanov J.M., Worline M.C. (2006). Seeing organizations differently: Three lenses on compassion. The SAGE Handbook of Organization Studies.

[25] Crant M.J. (2000). Proactive behavior in organizations. J. Manag..

[26] Seibert S.E., Crant J.M., Kraimer M.L. (1999). Proactive personality and career success. J. Appl. Psychol..

[27] Ashford S.J., Black J.S. (1996). Proactivity during organizational entry: The role of desire for control. J. Appl. Psychol..

[28] Major D.A., Kozlowski S.W. (1997). Newcomer information seeking: Individual and contextual influences. Int. J. Sel. Assess..

[29] Miller V.D., Jablin F.M. (1991). Information seeking during organizational entry: Influences, tactics, and a model of the process. Acad. Manag. Rev..

[30] Ostroff C., Kozlowski S.W. (1992). Organizational socialization as a learning process: The role of information acquisition. Pers. Psychol..

[31] Wanberg C.R., Kammeyer-Mueller J.D. (2000). Predictors and outcomes of proactivity in the socialization process. J. Appl. Psychol..

[32] Rhee S.-Y., Hur W.-M., Kim M. (2017). The relationship of coworker incivility to job performance and the moderating role of self-efficacy and compassion at work: The Job Demands-Resources (JD-R) Approach. J. Bus. Psychol..

[33] Baumgärtner M.K., Böhm S.A., Dwertmann D.J. (2014). Job performance of employees with disabilities: Interpersonal and intrapersonal resources matter. Equal. Divers. Incl. Int. J..

[34] Burns T., Catty J., Becker T., Drake R.E., Fioritti A., Knapp M., Lauber C., Rössler W., Tomov T., Van Busschbach J. (2007). The effectiveness of supported employment for people with severe mental illness: A randomised controlled trial. Lancet.

[35] Wrzesniewski A., Dutton J.E. (2001). Crafting a job: Revisioning employees as active crafters of their work. Acad. Manag. Rev..

[36] Tims M., Bakker A.B., Derks D. (2015). Job crafting and job performance: A longitudinal study. Eur. J. Work Organ. Psychol..

[37] Bakker A.B., Demerouti E. (2007). The job demands-resources model: State of the art. J. Manag. Psychol..

[38] Bakker A.B., Demerouti E., Verbeke W. (2004). Using the job demands-resources model to predict burnout and performance. Hum. Resour. Manag..

[39] Demerouti E., Bakker A.B., Nachreiner F., Schaufeli W.B. (2001). The job demands-resources model of burnout. J. Appl. Psychol..

[40] Frost P., Dutton J., Worline M., Wilson A., Fineman S. (2000). Narratives of compassion in organizations. Emotions in Organizations.

[41] Kanov J., Maitlis S., Worline M., Dutton J., Frost P., Lilius J. (2004). Compassion in organizational life. Am. Behav. Sci..

[42] Blau P.M. (1964). Exchange and Power in Social Life.

[43] Carmeli A., Spreitzer G.M. (2009). Trust, connectivity, and thriving: Implications for innovative behaviors at work. J. Creat. Behav..

[44] Aboul-Ela G.M.B.E. (2017). Reflections on workplace compassion and job performance. J. Hum. Values.

[45] Weiss H.M., Cropanzano R. (1996). Affective events theory: A theoretical discussion of the structure, causes and consequences of affective experiences at work. Res. Organ. Behav..

[46] Fredrickson B.L. (2001). The role of positive emotions in positive psychology: The broaden-and-build theory of positive emotions. Am. Psychol..

[47] Avey J.B., Wernsing T.S., Luthans F. (2008). Can positive employees help positive organizational change? Impact of psychological capital and emotions on relevant attitudes and behaviors. J. Appl. Behav. Sci..

[48] Grant A.M., Ashford S.J. (2008). The dynamics of proactivity at work. Res. Organ. Behav..

[49] Seo M., Barrett L.F., Bartunek J.M. (2004). The role of affective experience in work motivation. Acad. Manag. Rev..

[50] Svejenova S. (2005). The path with the heart: Creating the authentic career. J. Manag. Stud..

[51] Madrid H.P., Totterdell P., Niven K., Barros E. (2016). Leader affective presence and innovation in teams. J. Appl. Psychol..

[52] Fredrickson B.L., Cohn M.A., Coffey K.A., Pek J., Finkel S.M. (2008). Open hearts build lives: Positive emotions, induced through loving-kindness meditation, build consequential personal resources. J. Personal. Soc. Psychol..

[53] Vacharkulksemsuk T., Fredrickson B.L. (2013). Looking Back and glimpsing forward: The broaden-and-build theory of positive emotions as applied to organizations. Adv. Posit. Organ. Psychol..

[54] Fredrickson B.L., Losada M.F. (2005). Positive affect and the complex dynamics of human flourishing. Am. Psychol..

[55] Matsuo M. (2020). The role of work authenticity in linking strengths use to career satisfaction and proactive behavior: A two-wave study. Career Dev. Int..

[56] Lazazzara A., Tims M., De Gennaro D. (2020). The process of reinventing a job: A meta–synthesis of qualitative job crafting research. J. Vocat. Behav..

[57] Berg J.M., Grant A.M., Johnson V. (2010). When callings are calling: Crafting work and leisure in pursuit of unanswered occupational callings. Organ. Sci..

[58] Tims M., Bakker A.B., Derks D. (2012). Development and validation of the job crafting scale. J. Vocat. Behav..

[59] Bakker A.B., Tims M., Derks D. (2012). Proactive personality and job performance: The role of job crafting and work engagement. Hum. Relat..

[60] Shin Y., Hur W.M., Choi W.H. (2020). Coworker support as a double-edged sword: A moderated mediation model of job crafting, work engagement, and job performance. Int. J. Hum. Resour. Manag..

[61] Rudolph C.W., Katz I.M., Lavigne K.N., Zacher H. (2017). Job crafting: A meta-analysis of relationships with individual differences, job characteristics, and work outcomes. J. Vocat. Behav..

[62] Tajfel H. (1974). Social identity and intergroup behaviour. Soc. Sci. Inf..

[63] Tajfel H. (1975). The exit of social mobility and the voice of social change: Notes on the social psychology of intergroup relations. Soc. Sci. Inf..

[64] Bono J.E., Ilies R. (2006). Charisma, positive emotions and mood contagion. Leadersh. Q..

[65] Morrison E.W., Phelps C.C. (1999). Taking charge at work: Extrarole efforts to initiate workplace change. Acad. Manag. J..

[66] Williams L.J., Anderson S.E. (1991). Job satisfaction and organizational commitment as predictors of organizational citizenship and in-role behaviors. J. Manag..

[67] Baroudi S.E., Khapova S.N., Benlamri R., Sparer M. (2017). The effects of age on job crafting: Exploring the motivations and behavior of younger and older employees in job crafting. Leadership, Innovation and Entrepreneurship as Driving Forces of the Global Economy.

[68] Kooij D.T.A.M., van Woerkom M., Wilkenloh J., Dorenbosch L., Denissen J.J.A. (2017). Job crafting towards strengths and interests: The effects of a job crafting intervention on person–job fit and the role of age. J. Appl. Psychol..

[69] Becker T.E. (2005). Potential problems in the statistical control of variables in organizational research: A qualitative analysis with recommendations. Organ. Res. Methods.

[70] Becker T.E., Atinc G., Breaugh J.A., Carlson K.D., Edwards J.R., Spector P.E. (2016). Statistical control in correlational studies: 10 essential recommendations for organizational researchers. J. Organ. Behav..

[71] Spector P.E., Brannick M.T. (2011). Methodological urban legends: The misuse of statistical control variables. Organ. Res. Methods.

[72] Podsakoff P.M., MacKenzie S.B., Lee J.Y., Podsakoff N.P. (2003). Common method biases in behavioral research: A critical review of the literature and recommended remedies. J. Appl. Psychol..

[73] Williams L.J., Hartman N., Cavazotte F. (2010). Method variance and marker variables: A review and comprehensive CFA marker technique. Organ. Res. Methods.

[74] Preacher K.J., Hayes A.F. (2008). Asymptotic and resampling strategies for assessing and comparing indirect effects in multiple mediator models. Behav. Res. Methods.

[75] Shrout P.E., Bolger N. (2002). Mediation in experimental and nonexperimental studies: New procedures and recommendations. Psychol. Methods.

[76] Matsuo M. (2021). Reflection on success in promoting authenticity and proactive behavior: A two-wave study. Curr. Psychol..

[77] Quoidbach J., Mikolajczak M., Gross J.J. (2015). Positive interventions: An emotion regulation perspective. Psychol. Bull..

[78] Chatterjee S., Chakraborty S., Fulk H.K., Sarker S. (2021). Building a compassionate workplace using information technology: Considerations for information systems research. Int. J. Inf. Manag..

[79] Eldor L. (2018). Public service sector: The compassionate workplace—The effect of compassion and stress on employee engagement, burnout, and performance. J. Public Adm. Res. Theory.

[80] Miller K.I. (2007). Compassionate communication in the workplace: Exploring processes of noticing, connecting, and responding. J. Appl. Commun. Res..

[81] Tehan M. (2007). The compassionate workplace: Leading with the heart. Illn. Crisis Loss.

[82] Slemp G.R., Kern M.L., Vella-Brodrick D.A. (2015). Workplace well-being: The role of job crafting and autonomy support. Psychol. Well-Being.

[83] Coetzee S.K., Klopper H.C. (2010). Compassion fatigue within nursing practice: A concept analysis. Nurs. Health Sci..

[84] Figley C.R. (2002). Compassion fatigue: Psychotherapists’ chronic lack of self care. J. Clin. Psychol..

[85] Sabo B.M. (2006). Compassion fatigue and nursing work: Can we accurately capture the consequences of caring work?. Int. J. Nurs. Pract..

[86] Yoder E.A. (2010). Compassion fatigue in nurses. Appl. Nurs. Res..

[87] Ryan R.M., Deci E.L. (2000). Self-determination theory and the facilitation of intrinsic motivation, social development, and well-being. Am. Psychol..

[88] Bakker A.B., Demerouti E., Sanz-Vergel A. (2023). Job demands–resources theory: Ten years later. Annu. Rev. Organ. Psychol. Organ. Behav..

[89] Caniëls M.C., Baaten S.M. (2019). How a learning-oriented organizational climate is linked to different proactive behaviors: The role of employee resilience. Soc. Indic. Res..

[90] Parker S.K., Collins C.G. (2010). Taking stock: Integrating and differentiating multiple proactive behaviors. J. Manag..

[91] Dominguez L.C., Dolmans D., de Grave W., Sanabria A., Stassen L.P. (2019). Job crafting to persist in surgical training: A qualitative study from the resident’s perspective. J. Surg. Res..

[92] Harbridge R., Ivanitskaya L., Spreitzer G., Boscart V. (2022). Job crafting in registered nurses working in public health: A qualitative study. Appl. Nurs. Res..

[93] Buonocore F., de Gennaro D., Russo M., Salvatore D. (2020). Cognitive job crafting: A possible response to increasing job insecurity and declining professional prestige. Hum. Resour. Manag. J..

[94] Krause V., Rousset C., Schäfer B. (2023). Uncovering paradoxes of compassion at work: A dyadic study of compassionate leader behavior. Front. Psychol..

[95] Stevens K., Al-Abbadey M. (2023). Compassion fatigue and global compassion fatigue in practitioner psychologists: A qualitative study. Curr. Psychol..

